# The Role of the Gut Microbiome in Urinary Tract Infections: A Narrative Review

**DOI:** 10.3390/nu16213615

**Published:** 2024-10-24

**Authors:** Zaryan Safdar Iqbal, Sofie Ingdam Halkjær, Khaled Saoud Ali Ghathian, Julie Elm Heintz, Andreas Munk Petersen

**Affiliations:** 1 Gastrounit, Medical Section, Copenhagen University Hospital—Amager and Hvidovre, 2650 Hvidovre, Denmark; zaryan99@gmail.com (Z.S.I.); sofie.ingdam.halkjaer@regionh.dk (S.I.H.); 2 Department of Clinical Microbiology, Copenhagen University Hospital—Amager and Hvidovre, 2650 Hvidovre, Denmark; khaled.saoud.ali.ghathian@regionh.dk (K.S.A.G.); julie.elm.heintz@regionh.dk (J.E.H.); 3 Department of Clinical Medicine, University of Copenhagen, 2200 Copenhagen, Denmark

**Keywords:** gut microbiome, urinary tract infection, dysbiosis, short-chain fatty acids, *Escherichia coli*

## Abstract

Background/Objectives: Urinary tract infections (UTIs) represent a substantial health concern worldwide. Although it is known that the gut can act as a reservoir for UTI-causing pathogens, the exact role of the gut microbiome in developing UTIs remains unclear. This review aims to investigate the link between the gut microbiome and UTIs and whether gut dysbiosis increases the risk of getting a UTI. Methods: To find relevant studies, a search was conducted across three databases, PubMed, EMBASE and Cochrane Library. Only records that directly described the association between the gut microbiome and UTIs were included in this review. Results: Of the numerous studies retrieved, eight studies met the pre-set criteria and were selected for the review. The findings suggest several potential ways in which gut dysbiosis might enhance UTI susceptibility. A low gut microbiome diversity, a reduced level of bacteria involved in short-chain fatty acid (SCFA) production and a high abundance of *Escherichia coli* (*E. coli*) among UTI patients all offer a reasonable explanation for the existence of a link between an altered gut microbiome and UTIs. However, contradictory study results make it difficult to verify this. Conclusions: Research on the link between the gut microbiome and UTIs is limited, and further studies need to be carried out to substantiate this relationship, as this can bring attention to finding improved and more relevant treatment for UTIs.

## 1. Introduction

Urinary tract infections (UTIs) stand among the most prevalent bacterial infections globally, impacting about 150 million people yearly [1]. Common symptoms of UTIs include frequent urination, a burning sensation during urination, lower abdominal pain, and fever. Gender, age, sexual activity, previous UTIs and urinary tract abnormalities are all major risk factors associated with UTIs [2,3]. However, another factor that may contribute is the gut microbiome. Gut dysbiosis, a disruption in the balance of the gut’s microorganisms, is an area of active investigation. Emerging research suggests a connection between this imbalance and conditions affecting distant organs [4]. For instance, studies have discovered a potential relationship between gut dysbiosis and neurological conditions such as Parkinson’s disease and Alzheimer’s disease (gut–brain axis) [5]. Moreover, research indicates that a gut–kidney axis also exists, as an alteration in the gut microbiome has been linked with chronic kidney disease and kidney stones [6]. Several clinical studies have already shown that the intestine contributes to developing UTIs because the gut can act as a reservoir for UTI-causing pathogens, such as *Escherichia coli* (*E. coli*) [7,8]. UTIs commonly affect females due to anatomical factors. It typically begins with the contamination of the periurethral space by uropathogens residing in the gut. This is then followed by a colonisation of the urethra and an ascending migration to the bladder. Nevertheless, the existence of a gut–bladder axis is still being investigated [1,7,8].

There are various reasons why there might be a link between the gut microbiome and UTIs. Gut dysbiosis can contribute to an inflammatory state in the intestine [9]. An imbalance in the types and proportions of gut bacteria may trigger an inappropriate immune response, leading to chronic inflammation. In addition, a disrupted gut microbiome can compromise the integrity of the intestinal mucosal barrier, leading to increased permeability (leaky gut). This allows bacteria, toxins, and other molecules to pass through the gut lining and into the bloodstream to potentially trigger immune responses or infection elsewhere [10,11]. Additionally, dysbiosis involves alterations in the abundance and diversity of gut microbial species. Certain bacteria play a role in maintaining a balanced gut environment. If this balance is disrupted, overgrowth of specific bacterial groups may occur, which may lead to an intestinal bloom of potentially pathogenic bacteria [12,13].

Under normal conditions, certain genera of bacteria within the gut microbiome, such as *Bacteroides*, *Faecalibacterium*, and *Roseburia*, produce short-chain fatty acids (SCFAs) through the fermentation of dietary fibers. They primarily produce three main types of SCFA: acetate, propionate, and butyrate [14,15]. Intestinal dysbiosis can lead to an altered production of microbial metabolites, including a reduction in SCFAs [16]. This reduction can potentially affect the urinary tract in several ways. SCFAs are crucial for maintaining the health of the gut barrier and promoting normal intestinal motility. Moreover, SCFAs contribute to the defence against pathogenic bacteria by creating an environment that is unfavourable for their growth, e.g., by reducing the pH and modulating the expression of virulence factors. Additionally, SCFAs have immunomodulatory effects, help regulate inflammation and promote immune tolerance [17]. With these known effects, a lower level of SCFAs in the gut might enhance susceptibility to UTIs.

The standard treatment for UTIs is antibiotics [18]; however, frequent antibiotic consumption can have serious consequences. The overuse of antibiotics is a major driver of antibiotic resistance, a growing global health problem highlighted as a key challenge by the World Health Organization [19]. Antibiotic resistance complicates infection treatment, leading to longer illness durations, more complications, and in severe cases, the ineffectiveness of standard treatments [19]. Besides this, several studies have demonstrated that antibiotics can trigger gut dysbiosis [20,21]. If gut dysbiosis impacts UTI susceptibility, treatment using antibiotics can become a vicious cycle. Therefore, it is important to understand the role of the gut microbiome in preventing the recurrence of UTIs (rUTIs) and reducing unnecessary antibiotic use. This review aims to investigate the link between the gut microbiome and UTIs and to determine whether gut dysbiosis increases the risk of developing UTIs.

## 2. Methods

For this narrative review, the articles included were selected from searches performed in PubMed, Cochrane Library, and EMBASE on 30 August 2024. The search strategy included MeSH terms combined with free-text word terms. The free-text word terms were conducted using “all-fields” terms to ensure that the most recent papers, including those that have not yet received a MeSH term, could be retrieved from the databases.

The search string used for PubMed and Cochrane Library was: (“Gastrointestinal microbiome” MeSH OR “Gastrointestinal microbiome” OR “Gut microbiome” OR “Intestine microbiome”) AND (“Urinary tract infection” MeSH OR “Urinary tract infection”). In EMBASE, the MeSH term for “Gastrointestinal microbiome” was mapped to the subject heading “Intestine flora”. Hence, in this database, “Intestine flora” and “Urinary tract infection” were used as focused MeSH terms alongside the same free-text word terms used for PubMed and Cochrane Library.

The records were selected based on title and abstract during the initial screening process. Secondly, a full-text screening was conducted using inclusion and exclusion criteria. The inclusion criteria were studies directly describing the relation between gut microbiome and UTIs. No restriction was placed on the date of publication. The exclusion criteria were non-English studies.

## 3. Results

A limited number of human studies have specifically investigated the relationship between the gut microbiome and UTIs, and no relevant animal or in vitro studies were identified. Through a literature search, eight studies were found to meet the pre-set criteria (Table 1).

In the literature search, two paediatric studies were discovered. One of these is by Paalanne et al. [22], who carried out a prospective case-control study to assess the link between the gut microbiome and the risk of UTI. The gut microbiomes of 37 paediatric patients with a UTI were compared to 69 healthy age- and sex-matched controls. To analyse the microbiome, stool samples were collected from both patients and controls. The researchers sequenced the bacterial 16S rRNA gene and clustered them into operational taxonomic units (OTUs). Linear discriminant analysis effect size (LEfSe) was then employed to assess the linear discriminant analysis (LDA) [22]. This computational tool was used to identify differentially abundant features between groups by providing both statistical significance and effect size estimates.

Paalanne et al. [22] could not find any significant difference in the relative abundance of *E. coli* in the UTI patients and the controls. Moreover, the diversity in the gut microbiome was found to be similar among the patients and the controls, and no significant difference in the number of OTUs was observed. However, their analysis identified 20 OTUs that varied in abundance. Among these, *Enterobacter* was more abundant in UTI patients (LDA score of >3), whereas *Peptostreptococcaceae* was more abundant in the controls (LDA score of >3) [22].

In 2019, Thänert et al. [23] carried out a pilot study consisting of 14 patients with UTIs caused by antimicrobial-resistant (AR) uropathogens. In the study, the researchers found that the gut is a reservoir for uropathogens as the same isolates were recovered from urine and stool samples from patients with UTIs. Moreover, Thänert et al. found that most of the AR *E. coli* isolates obtained in the rUTI patients were similar to the clones obtained in their initial UTI episodes, indicating that instances of rUTIs are often caused by the same strain [23].

Further, Thänert et al. [23] observed that rUTIs were frequently preceded by a temporary overgrowth (intestinal bloom) of uropathogens. By combining semiquantitative culturing with comparative genomics, an increase in uropathogen abundance, relative to the previously collected specimen, was seen when a UTI was diagnosed. However, at other times, these intestinal blooms were observed in the absence of infection [23].

In 2019, Magruder et al. [24] investigated the link between the gut microbiome and the risk of developing bacteriuria or a UTI. In a cohort of 168 kidney transplant recipients, stool samples and urine cultures were collected regularly. On these specimens, 16S rRNA gene deep sequencing was performed. To analyse the results, a Cox regression was executed [24]. Notably, the researchers could not find a significant association between a 1% relative gut abundance of *Enterococcus* and the future development of *Enterococcus* UTI. However, a 1% relative gut abundance of *Escherichia* was found to be linked with the future development of *Escherichia* UTI. To further support the concept of the gut–bladder axis, the *E. coli* strains identified in the gut had a close similarity to the *E. coli* strain found in the urine of the same individual [24].

A year later, Magruder et al. [25] published another paper using the same cohort of 168 kidney transplant recipients to evaluate their microbial profiles. The researchers discovered that a high abundance of *Faecalibacterium* and *Romboutsia* is significantly associated with a reduced risk of *Enterobacteriaceae* UTIs. The study results showed an inverse relationship between the two bacteria taxa and *Enterobacteriaceae* UTIs, meaning that a low abundance of *Faecalibacterium* and *Romboutsia* increases the risk of getting *Enterobacteriaceae* UTIs [25].

Magruder et al. [25] also explored whether the relative abundances of these taxa changed after the diagnosis of *Enterobacteriaceae* UTIs. The results showed no significant change in the relative abundances of these taxa between the closest specimen collected before the UTI and the first specimen collected after the UTI. Moreover, the researchers investigated the effects of antibiotics in the gut and found that antibiotic administration was associated with decreased relative abundance of both *Romboutsia* and *Faecalibacterium* [25].

Worby et al. [26] performed a clinical study on 15 women with a history of rUTIs and a matched cohort of 16 healthy women to investigate the link between gut dysbiosis and rUTIs. The researchers collected urine, blood, and faecal samples for analysis. In women with rUTIs, a lower relative abundance of *Firmicutes* and elevated levels of *Bacteroidetes* were found at the phylum level. Overall, gut microbiome richness was found to be significantly lower in the rUTI group compared to the controls. Several of the taxa that were decreased in the rUTI gut are particularly involved in SCFA production. The decreased taxes include *Faecalibacterium*, *Akkermansia*, *Blautia*, and *Eubacterium hallii* [26].

Notably, the gut microbiome in the patient group did not show a significant difference in the abundance of *E. coli* compared to the control group. Moreover, the diversity of the *E. coli* strains was examined, and it revealed comparable patterns of presence in both groups. In addition, the researchers investigated whether an intestinal bloom in *E. coli* relative abundance is an rUTI risk factor. Blooms were defined as *E. coli* relative abundance >10-fold higher than the intra-host mean. Among the samples gathered, 22 instances of *E. coli* blooms were observed. Nevertheless, elevated *E. coli* levels were not predictive of UTIs because none of the 22 instances occurred in the two weeks preceding UTIs [26]. In most cases, the *E. coli* strain causing a UTI matched the strain obtained from a rectal swab, indicating a pathway from the intestine to the bladder. Besides this, the researchers found that treatment with antibiotics failed to permanently clear UTI-causing strains from the gut [26].

In another paediatric study, Urakami et al. [27] investigated whether an abnormal gut microbiome during infancy is a risk factor for developing febrile UTI. Twenty-eight infants aged between three and eleven months diagnosed with the first episode of a febrile UTI were recruited for the research, and these patients were compared to 51 healthy age- and sex-matched infants. Samples of stools were collected to perform 16S rRNA gene sequencing [27].

Alpha diversity (species diversity) was calculated using the Shannon index and showed that the microbial diversity in the gut was significantly lower in the UTI group compared to the control group. Beta diversity (variation in species) was calculated using the Bray–Curtis dissimilarity and significant differences were found in the gut microbiota between the UTI and control groups. Moreover, the LEfSe algorithm was used to analyse variation in gut microbiome abundance. It was discovered that *Enterobacteriaceae* and *Escherichia-Shigella*, among others, were more abundant in the gut microbiome in UTI patients (LDA score of >4), whereas *Bacteroides fragilis* was more abundant in healthy controls (LDA score of >4) [27].

In 2024, Choi et al. [28] carried out a study consisting of 125 patients with UTIs caused by an antibiotic-resistant organism to evaluate the connection between uropathogen colonisation and rUTIs. A segment of this cohort was initially presented in a pilot study by Thänert et al. in 2019 [23]. Stool and urine samples were taken regularly from the patients to analyse the taxonomic composition and resistance genes. The gut microbiome profiles of the UTI cohort were compared against published healthy reference microbiomes to find differences [28].

The Kruskal–Wallis test was used to analyse alpha diversity. This test showed lower species richness in the UTI group compared to the healthy controls; however, the difference was insignificant. To calculate the beta diversity, the Bray–Curtis dissimilarity was used. This revealed significant differences in the variation in species between the UTI and healthy samples. Using linear mixed-effect models, eleven gut taxa were found to differ significantly at the genus level between UTI samples and healthy controls. Genera reduced in UTI samples included *Parasutterella*, *Akkermansia*, and *Bilophila*, whereas healthy controls showed an enrichment of commensal Firmicutes, such as *Ruminococcus*, *Roseburia*, and *Eubacterium* [28]. Additionally, Choi et al. [28] observed a significant reduction in gut microbiome species richness during and after antibiotic treatment.

In another study, Miller et al. [29] investigated the gut microbiome in aged care residents. Fifty-four patients with a history of UTIs were compared with 69 age- and sex-matched controls with no UTI history. The researchers found that the gut microbiome between the UTI and control group was not significantly different. Additionally, the alpha diversity did not differ significantly between the two groups. However, the analysis identified nine species that differed significantly between individuals with a prior UTI and those without. Among these, *Bifidobacterium dentium*, *Dorea longicatena*, and *Lactobacillus rogosae* were less abundant in the UTI group [29]. Interestingly, the gut microbiome in the UTI patient group did not display a notable difference in *E. coli* relative abundance compared to the control group.

As UTI incidence increases with age, Miller et al. [29] further compared a group of aged care residents with UTIs with a group of 20 younger adults without UTIs. This comparison revealed that the gut microbiome of aged care residents had significantly lower diversity and lower levels of SCFA-producing taxa, particularly taxa involved in butyrate production. Among the bacteria identified as significantly lower in abundance in the UTI group were *Bifidobacterium adolescentis*, *Faecalibacterium prausnitzii* and *Blautia wexlerae*. In addition, Miller et al. [29] found a significant association between prior antibiotic use and a change in gut microbiome. However, when the analysis was limited to UTI-exclusive antibiotics, no significant relationship was found.

## 4. Discussion

This review aimed to investigate the link between the gut microbiome and UTIs and to determine if gut dysbiosis increases the risk of developing a UTI. Only a limited number of studies are available on this subject, and differences between the study groups, study execution, and analysis make it difficult to compare results effectively. Some studies include children and others involve adults. Additionally, only a few studies include females, whereas others involve both males and females. These variations have an impact as age, sex, and environment can all affect the composition of the gut microbiome making it difficult to draw conclusions. However, although the direct relationship between the gut microbiome and UTIs is complex and still not fully understood, research suggests several potential ways in which gut dysbiosis might increase susceptibility to UTIs.

The current evidence suggests that the gut can act as a reservoir for uropathogens. Worby et al. [26], Magruder et al. [24] and Thänert et al. [23] all recovered the same isolates from urine and stool samples from patients with UTIs. These data indicate the existence of a microbial transmission axis connecting the gut and bladder.

The study results, furthermore, show a possible connection between gut dysbiosis and UTIs. The diversity of the gut microbiome seems to be lower in UTI patients compared to healthy cohorts, and this factor could increase UTI susceptibility. Although Paalanne et al. [22] and Miller et al. [29] found similar gut microbiome diversity between UTI patients and the controls, both Urakami et al. [27] and Worby et al. [26] found a significantly lower gut microbiome richness in UTI patients. Gut dysbiosis may indirectly contribute to UTIs through various mechanisms, such as an imbalance in immune responses [9,10]. However, even though Worby et al. [26] adjusted their results for recent antibiotic use, they could not rule out the possibility that a history of antibiotic use may have contributed to lower microbiome diversity. Furthermore, Urakami et al. [27] could not exclude the possibility that previous exposure to antibiotics in the UTI group had influenced microbiome diversity. Indeed, antibiotic treatment is a confounding factor in these studies, as differences in diversity potentially reflect the impact of UTI treatment rather than signalling elevated susceptibility to infections [20,21].

Some of the studies did investigate the influence of antibiotics on the gut microbiome. Magruder et al. [25], Choi et al. [28] and Miller et al. [29] all found a significant difference in the gut microbiome after the use of antibiotics. On the other hand, both Paalanne et al. [22] and Worby et al. [26] discovered that antibiotic exposure did not significantly change the gut microbiome during the study period. Nonetheless, the short study duration makes it difficult to exclude the possibility that repeated antibiotic exposure over the years still impacts the gut microbiome. The presented results appear to be contradictory, and whether gut dysbiosis is a direct result of long-term antibiotic exposure remains to be elucidated, and further studies need to be carried out.

A common finding across the studies is that UTI patients tend to have notably reduced levels of bacteria involved in SCFA production. Whereas Worby et al. [26] found low levels of *Faecalibacterium*, *Akkermansia*, *Blautia* and *Eubacterium hallii* in UTI patients, Paalanne et al. [22] found a low proportion of *Peptostreptococcaceae* in UTI patients and Urakami et al. [27] found a low proportion of *Bacteroides fragilis* in UTI patients. Moreover, Magruder et al. [25] discovered that a low abundance of *Faecalibacterium* and *Romboutsia* increased the risk of developing *Enterobacteriaceae* UTIs [25], and both Choi et al. [28] and Miller et al. [29] found a lower level of various bacterial taxa that are responsible for producing SCFAs in the gut. SCFAs play a crucial role in maintaining gut health and homeostasis by supporting the integrity of the intestinal barrier, reducing inflammation, and promoting mucus production. A decrease in the bacterial taxa responsible for producing SCFAs may disrupt these advantageous functions in the gut, potentially creating favourable conditions for uropathogens to infect the urinary tract [14,17]. Given the known effects of SCFAs, these data present a probable association between decreased immunomodulatory gut microbial taxa and UTIs.

Several bacteria can cause UTIs, but *E. coli* is the most predominant pathogen causing 80–90% of community-acquired UTIs [30]. Several of the studies investigated whether the gut richness of *E. coli* had relevance. Urakami et al. [27] discovered that the proportion of the genus *Escherichia-Shigella*, which includes *E. coli*, was significantly higher in the UTI group compared to the healthy group [27]. Moreover, Magruder et al. [24] observed that a 1% relative gut abundance of *Escherichia* was linked to the future development of *Escherichia* UTIs [24]. However, Paalanne et al. [22], Worby et al. [26] and Miller et al. [29] could not find any significant difference in the relative abundance of *E. coli* between UTI patients and controls.

Gut dysbiosis can create an environment favourable to a temporary overgrowth of certain bacteria, also known as an intestinal bloom. Research indicates that gut dysbiosis supports the evolution of pathogens by promoting the transfer of antibiotic resistance and virulence genes [12]. Both Worby et al. [26] and Thänert et al. [23] investigated whether an intestinal bloom in *E. coli* abundance is a risk factor for developing UTIs. Both studies observed *E. coli* blooms in gut dysbiotic patients; however, whereas Worby et al. [26] found that intestinal blooms were not predictive of UTIs, Thänert et al. [23] found that these blooms are sometimes linked to UTIs. Overall, the results are contradictory, and more studies are necessary to understand the link between the relative abundance of *E. coli*, intestinal blooms and UTIs.

Worby et al. [26] found that plasma eotaxin-1, a chemokine associated with intestinal inflammation, was higher in women with rUTIs compared to controls. Moreover, the researchers compared the gut microbiome of the rUTI patients in their study with data from the Human Microbiome Project 2 (HMP2) study, revealing that these patients shared similarities with individuals with chronic gut disorders like inflammatory bowel disease (IBD) [26,31]. Potential connections between gut dysbiosis and rUTIs could mean individuals with diseases like IBD also have an increased UTI risk due to similar microbiomes. However, this remains unresolved as relevant publications on this relationship could not be found.

In terms of UTI treatment, antibiotics are the frontline therapy [32]. Nevertheless, both Thänert et al. [23] and Worby et al. [26] observed that UTI-causing strains often persist in the gut despite antibiotic treatment. Moreover, it is uncertain whether antibiotics contribute to the formation of gut dysbiosis, and whether gut dysbiosis impacts UTI susceptibility. Potentially, antibiotics could be part of a vicious cycle where treatment increases the risk of developing a recurrence of infection. Avoiding antibiotics for a period may help the microbiome to regain a healthier state. Although the precise mechanisms remain unclear, the gut microbiomes’ influence on UTIs unveils potential targets for antibiotic-sparing treatment and prophylaxis. Faecal microbiota transplantation (FMT) is an encouraging indication of the potential success of microbiome-based therapeutics. Studies have shown that among *C. difficile* patients who underwent FMT, there was a decrease in the frequency of rUTIs [33,34]. Furthermore, in a case report, a patient with gut dysbiosis in the form of irritable bowel disease (IBS), experienced fewer UTIs and fewer IBS symptoms after going through FMT [35]. Moreover, in a study cohort consisting of five patients with UTIs caused by multidrug-resistant organisms, FMT was successful in lowering the incidence of UTIs and significantly reduced hospital expenses [36]. In addition to this, probiotics have also emerged as a promising microbiome-based therapy for treating UTIs. A recent randomized controlled trial involving 174 premenopausal women with rUTIs found that both oral and vaginal probiotics helped reduce the incidence of UTIs [37]. Nevertheless, the findings are inconsistent across studies. In a systematic review of nine studies, only two reported a significant reduction in the risk of UTIs associated with probiotic use [38]. Besides this, other treatments, such as phage therapy [39] and anti-adhesion molecules [40], have also indicated some positive outcomes in UTI treatment. Although, in general, microbiome-based therapeutics demonstrate mixed results, the ongoing research highlights significant potential. Therefore, directing more attention towards these non-antibiotic alternatives to prevent rUTIs provides a favourable path for future research.

## 5. Conclusions

Currently, it is challenging to verify whether there is a link between an altered gut microbiome and UTIs. Contradictory study results make it difficult to determine whether gut dysbiosis increases the risk of developing UTIs or whether it is a result of UTIs and repeated antibiotic treatment. A low gut microbiome diversity, a reduced level of bacteria involved in SCFA production and a high abundance of *E. coli* in the gut among UTI patients all offer reasonable explanations for the existence of a link between an altered gut microbiome and UTIs. Therefore, to prevent the recurrence of UTIs and to facilitate improved targeted treatment, further studies need to be carried out to substantiate this link.

## Figures and Tables

**Table 1:** Studies included in this review. Note, Magruder et al. published two studies using the same cohort.

Reference	Trial Design	Country	Microbiome Method	Sample Size	Median Age	Sex
Paalanne et al. [22], 2018	Case-control study	Finland	16S rRNA sequencing and qPCR	Case: n = 37 Control: n = 69	Case: 20.3 months Control: 21.8 months	Male: 28.3% Female: 71.7%
Thänert et al. [23], 2019	Prospective cohort study	United States	16S rRNA sequencing and WGS	Cohort: n = 14	Cohort: 63 years	Female: 100%
Magruder et al. [24], 2019 Magruder et al. [25], 2020	Prospective cohort study	United States	16S rRNA sequencing and shotgun metagenomic sequencing	Cohort: n = 168	Cohort: 55 years	Male: 54.8% Female: 45.2%
Worby et al. [26], 2022	Case-control study	United States	Shotgun metagenomic sequencing	Case: n = 15 Control: n = 16	Case: 28.3 years Control: 29.3 years	Female: 100%
Urakami et al. [27], 2023	Case-control study	Japan	16S rRNA sequencing	Case: n = 28 Control: n = 51	Case: 5 months Control: 5 months	Male: 53.1% Female: 46.8%
Choi et al. [28], 2024	Prospective cohort study	United States	Shotgun metagenomic sequencing	Cohort: n = 125	Cohort: 58 years	Male: 6.4% Female: 93.6%
Miller et al. [29], 2024	Case-control study	Australia	Shotgun metagenomic sequencing	Case: n = 54 Control: n = 69	Case: 87.6 years Control: 87.6 years	Male: 18.7% Female: 81.3%

qPCR: Quantitative polymerase chain reaction; WGS: whole-genome sequencing.

## Data Availability

All data generated and analysed during this study are included in this published article.
